# Visuomotor Memory Is Not Bound to Visual Motion

**DOI:** 10.1523/JNEUROSCI.1884-24.2025

**Published:** 2025-03-14

**Authors:** Matthew Warburton, Carlo Campagnoli, Mark Mon-Williams, Faisal Mushtaq, J. Ryan Morehead

**Affiliations:** ^1^School of Psychology, University of Leeds, Leeds LS2 9JT, United Kingdom; ^2^Bradford Institute for Health Research, Bradford BD9 6AF, United Kingdom; ^3^National Centre for Optics, Vision and Eye Care, University of South-Eastern Norway, Kongsberg 3616, Norway; ^4^Centre for Immersive Technologies, University of Leeds, Leeds LS2 9JT, United Kingdom

**Keywords:** generalization, learning, video games, visuomotor adaptation

## Abstract

The motor system adapts its output in response to experienced errors to maintain effective movement in a dynamic environment. This learning is thought to utilize sensory prediction errors, the discrepancy between predicted and observed sensory feedback, to update internal models that map motor outputs to sensory states. However, it remains unclear what sensory information is relevant (e.g., the extent to which sensory predictions depend on visual feedback features). We explored this topic by measuring the transfer of visuomotor adaptation across two contexts where input movements created visual motion in opposite directions by either (1) translating a cursor across a static environment or (2) causing the environment to move toward a static cursor (272 participants: 94 male, 175 female). We hypothesized that this difference in visual feedback should engage distinct internal models, resulting in poor transfer of learning between contexts. Instead, we found nearly complete transfer of learning across contexts, with evidence that the motor memory was bound to the planned displacement of the hand rather than visual features of the task space. Our results suggest that internal model adaptation is not tied to the exact nature of the sensory feedback that results from movement. Instead, adaptation relies on representations of planned movements, allowing a common internal model to be employed across different visual contexts.

## Significance Statement

Human motor control requires constant calibration to remain effective in a dynamic environment. This adaptive process is thought to be driven by error-based learning in internal models that either predict the sensory consequences of a planned movement or output the required movement to realize a sensory goal. However, what sensory information is relevant is unclear. We probed whether internal model adaptation, in response to rotated visual feedback, transferred across two contexts where a common hand movement caused visual motion in opposite directions. We found near-complete transfer of learning across these two contexts and that learning was tied to hand movements. These results indicate that internal models operate at a level abstracted from the exact nature of the visual feedback provided.

## Introduction

Internal models are central to current theories of motor control and learning (Shadmehr and Krakauer, 2008; Franklin and Wolpert, 2011; Krakauer et al., 2019; McNamee and Wolpert, 2019). Forward models are thought to be useful for online control (Miall and Wolpert, 1996; Mehta and Schaal, 2002), robust state estimation (Desmurget and Grafton, 2000), and attenuating self-generated sensory signals (Blakemore et al., 2000; Sawtell, 2017) by predicting the sensory consequences of a motor command. Controllers, or inverse models, do the opposite: they generate the motor commands needed to achieve a sensory outcome (Jordan and Rumelhart, 1992) and can operate as multiple forward-inverse internal model pairs across different contexts (Wolpert and Kawato, 1998; Haruno et al., 2001). The specific features that make sensory information relevant for these internal models, and therefore what sensory feedback changes might delineate context-specific internal models, is generally unclear.

**Figure 1.**
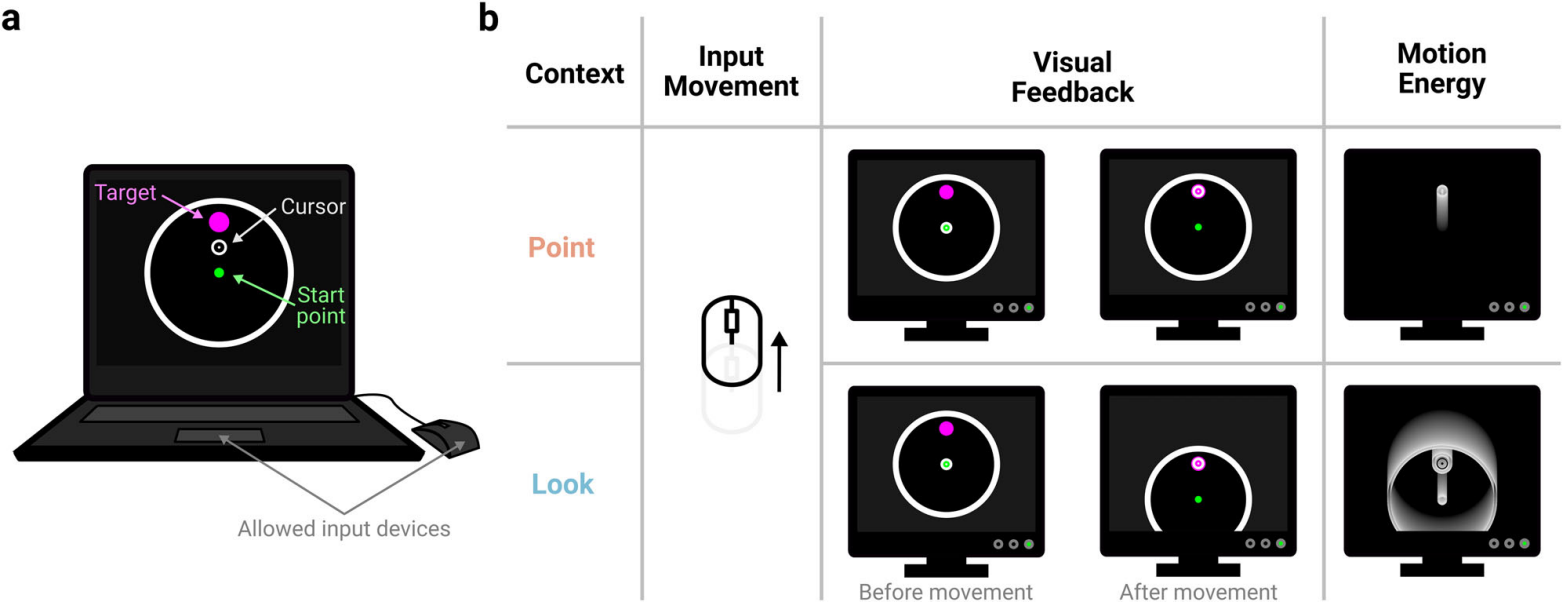
Task setup. ***a***, A visual representation of the task as seen by a participant in the Point mode. Participants used their personal computers and could interact with the task using either a trackpad or computer mouse. Participants left-clicked a start-point and were shown one of four targets to move to. Participants attempted to move to and left-click the target within a continuously staircased time limit. ***b***, Differences between the contexts, illustrated for a common “up” movement of the input device. In the Point mode, input movements translate the cursor across a static background. In the Look mode, the same input movements cause the view of the virtual environment to shift, giving visual feedback where everything except the cursor moves. This gives rise to oppositely directed visual motion energy as the movement evolves over time.

This lack of clarity is felt when considering behaviors that differ only in their visual feedback. For example, when moving a computer mouse to highlight a specific word in a text document, we might be surprised to find that the text moved toward the cursor. It is unclear whether such visual differences matter to the motor system. We recently tested an analogous scenario by comparing a common reaching task where movement-contingent visual feedback varied between two contexts (Warburton et al., 2023). In a Pointing context, a forward-directed hand movement would translate a cursor upwards across static task objects. In a Looking context, inspired by first-person shooter (FPS) video games, the same input movement would tilt the view of the task upward, effectively translating everything (except a centrally fixed cursor) downward across the screen. Thus, while a given target can be acquired by a common reaching movement in either context, the visual feedback experienced is oppositely directed (Fig. 1, motion energy panel). Interestingly, within-subject task performance and movement kinematics were highly correlated across contexts, suggesting they may rely on shared internal models.

Here we sought to more directly test whether the two visual contexts engage the same internal models by assessing the transfer of visuomotor adaptation between visual contexts. When the usual mapping between a movement and its sensory outcome is perturbed, such as introducing a rotation between hand and cursor movements, future movements are adapted to minimize the discrepancy (Shadmehr et al., 2010; Krakauer et al., 2019; Kim et al., 2021), a process thought to rely on sensory prediction errors (Mazzoni, 2006; Tseng et al., 2007). To remain useful for effective control, internal models must be continuously calibrated, and work has posited that adaptation is driven by updates to a forward model (Shadmehr et al., 2010; Krakauer and Mazzoni, 2011), a controller (Raibert, 1978; Hadjiosif et al., 2021), or both (Bhushan and Shadmehr, 1999). Critically, the observation of poor learning transfer would suggest that the adapted internal model is tied to the specific style of visual feedback presented.

To investigate the extent to which visuomotor adaptation is bound to specific features of the visual feedback, we introduced a 30° rotation between a reaching movement and the visual feedback of the reach, requiring that participants adapt their movements to maintain accuracy (Cunningham, 1989; Krakauer et al., 2000). We hypothesized that the distinct visual feedback in the two contexts would engage separate internal models, independently adapted, leading to limited transfer of learning. First, we compared adaptation in either context, where we might expect differences in the learning response if distinct internal models are adapted. Next, we assessed transfer of learning from one trained context to the other (untrained) one, where we would expect poor transfer of learning if distinct adapted internal models are engaged. Finally, we dissociated hand and visual movement vectors to assess which feature was bound to learning.

## Materials and Methods

### Participants

In total, 276 participants were recruited to take part in the experiments. After exclusions (*n* = 2 in Experiment 2: 1 participant recorded no successful movements after the first block, 1 participant's data did not upload; *n* = 2 in Experiment 3: participant data did not upload), the final sample was 272 participants. Participants were recruited through Prolific.co, an online recruitment platform, and were restricted to those living in the UK or USA, who had English as a first language, and had a Prolific approval rating of 95% or above. Participants were paid between £6.25 and £8 upon completion for expected completion times between 50 and 60 min depending on experiment. Experiments were approved by the School of Psychology Ethics Committee at the University of Leeds, and participants gave informed consent via a Web form prior to starting the study. Given the experiment was completed online, which has been associated with noisier responses within and between participants on similar paradigms (Tsay et al., 2021), we recruited more participants per group (between 30 and 39) than comparable laboratory studies (Sheahan et al., 2016; Abekawa et al., 2022; Dawidowicz et al., 2022). Specifically, we aimed to recruit a typical sample from previous experiments per input device, so we continued recruiting until we had at least *n* = 15 per input device per experimental group.

### Apparatus and experimental procedure

Participants completed experiments using their own personal computer (restricted by Prolific's screening criteria to those using a laptop or desktop) and completed reaching movements in the game using a mouse or trackpad. In the main text, we present data from both mouse and trackpad, as we did not observe any meaningful differences between these input devices. Experiments were created using a recently developed framework allowing within- and between-participant comparison of Pointing and Looking movements (Warburton et al., 2023), which uses the Unity game engine (2019.4.15f) and the Unity Experiment Framework (Brookes et al., 2019). The experiments were delivered via a WebGL build hosted on a Web page. No calibration procedure was performed to equate stimuli size across disparate participant monitor dimensions, so the physical size of the task was not constant across participants. The experiments were designed to be visible on a 4:3 monitor aspect ratio, with the height always taking up 4 arbitrary Unity units (au) and wider aspect ratios featuring more of a task-irrelevant background texture. The experiments could only be completed in full-screen mode with the desktop cursor locked and hidden, with raw mouse or trackpad input used to perform in-game movements.

Participants first filled in a form to provide details on age, gender, handedness, computer type, and input device (mouse or trackpad) and also clicked a button to ensure game audio was audible. During this stage, participants used their desktop cursor to navigate the form, and the movements of this cursor were tracked in pixel and Unity game units to provide an initial calibration for the in-game cursor. Participants were shown a cut-scene providing exposition for the purpose of the study (popping nonsentient space bubbles), before completing a tutorial that introduced aspects of the experimental task sequentially and interactively to ensure participants fully understood how to complete the experiment. Following this, participants were required to practice using both contexts used in the experiment (Point and Look for Experiments 1–3, or Point and Inverted Look for one group in Experiment 3), where they could switch contexts and adjust cursor sensitivity with key presses. Once participants had completed at least 20 trials in each context, they could progress to the main task.

Across all experiments, participants used their mouse or trackpad to move an FPS style cursor toward a presented target and shoot it (Fig. 1*a*). Throughout a trial, participants saw a dark space-themed background. On top of this, the target plane was shown, which had a diameter of 3 au, a black background, and a thin ring around it which was white for Experiments 1 and 2 and either yellow or cyan for Experiment 3. When a trial was ready to start, the start-point (0.1 au diameter circle centered on the target plane) was colored orange. Participants needed to move their in-game cursor, a small white circle surrounded by a thin white ring of diameter 0.15 au (to mimic FPS style reticles), to the start-point and left-click it to start a trial. If participants were within 0.05 au of the start-point during this homing movement, the cursor snapped to the start-point to speed up this centering (mimicking FPS-style auto-aim features). Once the start-point had been clicked, a target (0.2 au diameter magenta circle) immediately appeared on the target plane, and the start-point turned green to indicate participants could attempt to move to and left-click the target to shoot it. Potential targets were located in increments of 90° on an imaginary circle of diameter 2au. For Experiments 1 and 2, participants had a time limit within which to shoot the target. If the time limit elapsed without a successful shot being registered, the target immediately vanished and a whooshing sound was played. A shot was only successful if, at the time of the left-click, the center of the cursor intercepted the target circle. If the shot was successful, the target exploded and a shooting sound was played. Unsuccessful shots had no effect upon the game, and an unlimited number of attempted shots were allowed per trial. Feedback of a trial outcome, either a successful shot or a lapsed time limit, was provided for 300 ms, during which the start-point was colored gray. Upon feedback finishing, the start-point turned orange, indicating that a new trial could begin. Participants had online feedback of the cursor throughout a trial, apart from Experiment 3, where certain trials had no feedback when returning to the start-point.

Cursor movements could be performed in two main contexts, either the Point or Look (Fig. 1, with an Inverted Look context added for Experiment 3, Fig. 4*a*). In either context, a viewport camera provided a display of the virtual environment, shown on the participant's monitor, on top of which a cursor was rendered. In the Point context, the camera remained static throughout a trial, and input movements of the mouse or trackpad translated the cursor across this static display. In the Look and Inverted Look contexts, input movements panned and tilted the camera's view of the scene while the cursor remained fixed to the center of the camera's view. This has the visual effect of a target being pulled toward the cursor, located in the center of the screen. Critically, the two contexts were equated to ensure identical movements were required to reach a given target in either context. Details of the implementation are described in a previous paper (Warburton et al., 2023). Following completion of the experimental trials, participants filled in a questionnaire probing their perception of the experiment's performance (e.g., lag, difficulty, technical issues), as well as questions about strategy use to overcome the imposed perturbation.

#### Experiment 1

Participants (*n* = 68; mean age = 36, SD age = 11, age range = 20–65; 24 male, 42 female) were assigned to complete the experiment in either the Point (*n* = 32) or Look (*n* = 36) context. During the main task, participants completed 680 experimental trials. Participants first completed a baseline period of 100 experimental trials where no perturbation was applied to cursor movements. Following this, participants completed 480 experimental trials with a 30° visuomotor rotation, where the cursor movement was rotated away from the actual input movement in either the clockwise or counterclockwise direction (counterbalanced across participants within each group). Finally, participants completed 100 experimental trials where the perturbation was turned off to assess after-effects of learning from the rotation period. Throughout the experiment, trials were tested in cycles of four trials, where each target (0, 90, 180, or 270°) was tested in a random order. Participants were given breaks of unrestricted length every 20 cycles through the experiment.

To induce the type of time pressure that might be experienced in a typical FPS-style game, the time limit within which participants had to show a target was continuously staircased throughout the experiment. A pair of staircases, initiated at 450 and 1,050 ms for mouse users or 780 and 1,380 ms for trackpad users [determined through previous work (Warburton et al., 2023)], were interleaved and used equally within each block to set the time limit for the upcoming trial. Following a 1-up 1-down procedure, the tested staircase's time limit was decreased by 30 ms following a successful trial, and increased by 30 ms following an unsuccessful trial, to approximately give a 50% success rate over the course of the experiment.

#### Experiment 2

Participants were assigned to either Experiment 2a (*n* = 62; mean age = 37, SD age = 11, age range = 18–67; 24 male, 38 female) or Experiment 2b (*n* = 66; mean age = 36, SD age = 11, age range = 20–75; 20 male, 46 female) and were further assigned to complete the rotation block in either the Point (Experiment 2a, *n* = 32, Experiment 2b, *n* = 35) or Look (Experiment 2a, *n* = 30, Experiment 2b, *n* = 31) context. The experiment consisted of 800 trials. Participants first completed 100 baseline trials in the “untrained” context (the Look context for the Point Trained group) and then 100 baseline trials in the “trained” context (the Point context for the Point trained group). During these trials, no perturbation was applied to the cursor. Following this, participants completed 480 trials in the trained context with a 30° visuomotor rotation (direction counterbalanced within each group). For Experiment 2a, the perturbation was then turned off, and participants completed 100 trials in the untrained context and a further 20 trials in the trained context, to assess the transfer of learning from the trained to untrained context. Participants in Experiment 2b completed the same pair of blocks after the rotation, but the perturbation was left on during these blocks, as a second method of assessing transfer. As in Experiment 1, trials were tested in cycles of the four target directions, and the time limit was continuously staircased throughout to induce time pressure. Participants were given untimed breaks after cycles 23 and 45 and thereafter every 20 cycles.

In Experiment 1, it was observed that participants sometimes initiated movements to an uncued target (hereafter “jump starts”). To combat this, a further trial outcome was added. If a participant's movement at a radius of 0.2 au was >60° in either direction from the ideal movement path between the start-point and target, the trial immediately stopped, and participants were shown an error message for 5 s instructing them to make sure they waited to see the target before moving. This check was performed early enough that participants only received a limited sample of their movement on these failed trials. The trial was then repeated, to ensure that participants all received the same number of trials to assess learning and transfer.

#### Experiment 3

Participants (*n* = 76; mean age = 36, SD age = 13, age range = 19–67; 26 male, 49 female) were assigned to use either the Look (*n* = 40) or Inverted Look (*n* = 36) context during probe trials. In total, participants completed 760 trials during this experiment. In contrast to the previous experiments, no time limit was used, as pilot testing found this caused a substantial number of errors on the critical probe trials. Further, the ring around the target plane changed color as participants returned their cursor to the start-point to indicate the context of the upcoming trial.

Participants first completed 60 baseline trials to the top and bottom targets (90° and 270°). The first 40 trials had 20 contiguous trials for each tested context (Point and either Look or Inverted Look, in a random order), followed by 20 trials where the two contexts were interleaved to get participants used to switching between contexts. Participants then completed 20 trials in the Point context to the top target, where the no-feedback return was introduced. Here, upon clicking the target, the cursor disappeared, and participants were required to return their hand to a comfortable position within 1.5 s of outcome feedback extinguishing. Tones were played after 500, 1,000, and 1,500 ms to ensure participants knew when their hand had to be back to a comfortable position. After the final tone, the cursor appeared in the middle of the start-point. From this trial onward, Point trials never had return feedback (whereas Look and Inverted Look trials always had return feedback) and were always directed to the 90° (top) target.

Participants then completed the baseline generalization block, an unperturbed version of the generalization block. The baseline generalization block consisted of 15 generalization cycles of 18 trials. The first three trials were in the probed context, either Look or Inverted Look, and probed participants movements to either the left or right target (0 or 180°) in the first trial and both the top and bottom targets in trials two and three (arranged so the top target was tested first either seven or eight times, in a random order across cycles). The first movement to a lateral target served as an additional cue, as well as the ring color, that the context had switched before the critical vertical targets were probed. The last 15 trials per cycle were in the Point context to the top target. During the baseline generalization block, movements were unperturbed compared with their nominal mapping. Participants then performed 120 trials in the Point context with a 30° visuomotor rotation applied (rotation direction counterbalanced within each group). Because there was no return feedback, participants only sampled learning during movements to the top target, so should show only local generalization. Participants then completed the generalization block, which had the same structure as the baseline generalization block except the 15 Point trials per cycle had the same visuomotor rotation as in the rotation block (while the probe trials were never perturbed). Because the probe trials had return feedback and probed both trained and untrained directions, unlearning could occur during outward and return reaches, so these 15 trials “topped up” learning to ensure the probes were made after learning again reached asymptotic levels, as determined during pilot testing. While generalization studies typically preclude feedback to avoid this issue, the feedback during Look trials arose because the camera panned and tilted, so clamping the camera's rotation during Look or Inverted Look probes would give no indication a Look style movement had theoretically occurred. Participants finally completed 20 Point movements with unperturbed feedback, to assess after-effects. Participants were given breaks of unrestricted length during the 8th probe cycle in both the baseline generalization and generalization blocks (after the probe trials but before the top-up trials) and after 100 trials in the rotation block.

The same online check for jump-starts used in Experiments 2a and 2b was used here for trials during the first baseline block for both contexts and thereafter only for the Point context. As we expected some level of “slips” for the Inverted Look group (i.e., expressions of the non-inverted mapping), we did not check for jump-starts during the probe trials.

### Data analysis

Kinematic data (cursor position in *x-* and *y*-axis) was sampled at the participant's screen refresh rate (often approximately 60, 120, or 144 Hz) and uploaded to a remote database during the experiments. Offline processing was performed in R (version 4.2.2). Technical issues meant a small number of trials were not uploaded, which removed 0.02% of trials in Experiment 1, 0.05% of trials in Experiment 2, and 0.02% of trials in Experiment 3. Further, a small number of trials were affected by an issue that caused a large single-frame mouse input, causing participant's cursor to move a large distance suddenly and unexpectedly. This appears to be an issue with WebGL games more broadly, so we could only filter out affected trials post hoc by removing trials where cursor speed changed by >40 au/s from a previous or following sample. This removed 0.4% of trials in Experiment 1, 0.04% of trials in Experiment 2, and 0.3% of trials in Experiment 3.

**Figure 2.**
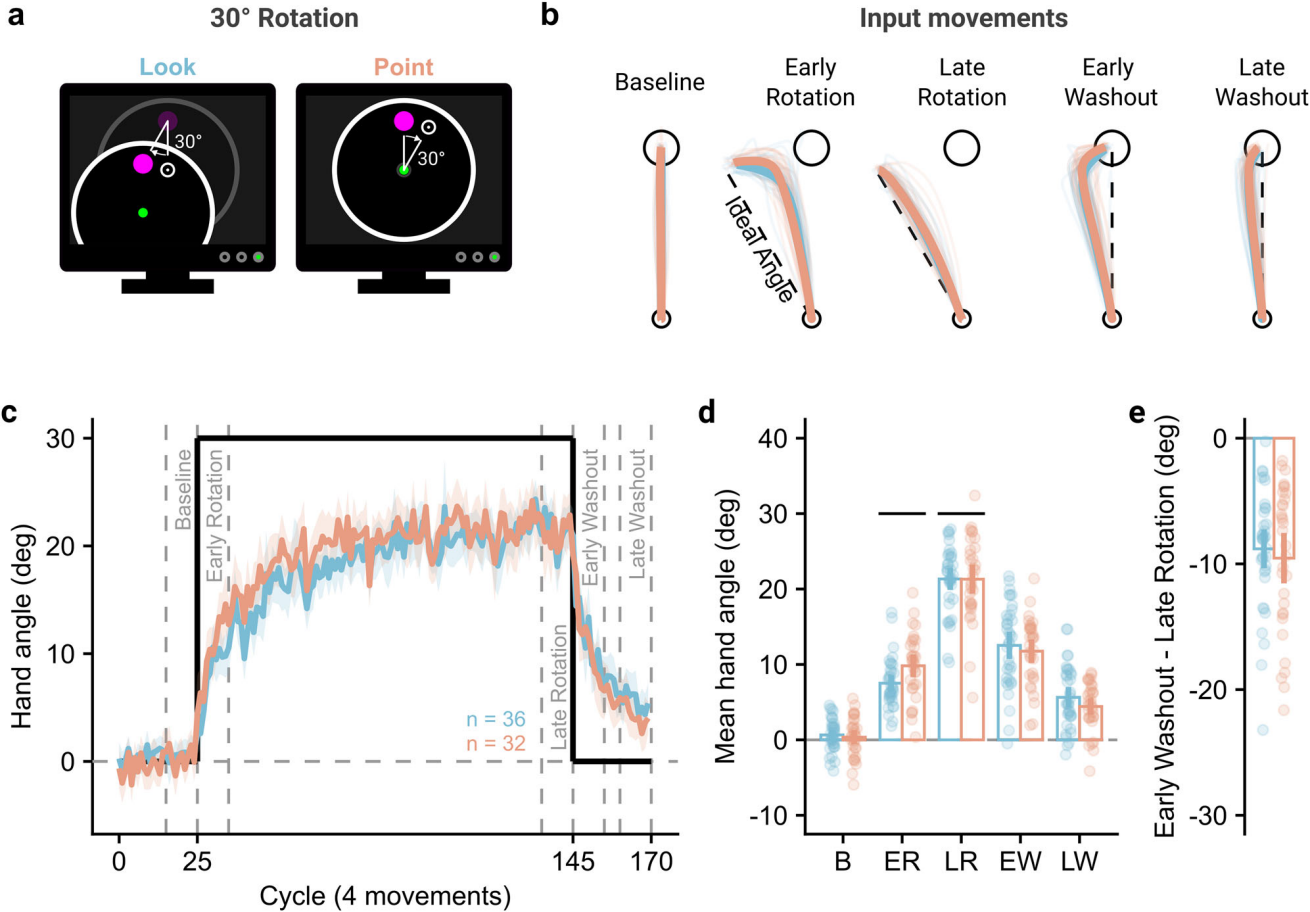
Participants adapted similarly across contexts. ***a***, During Experiment 1, participants (*n* = 68) experienced a visuomotor rotation that meant in-game cursor movements were rotated 30° from the veridical movement direction. ***b***, Participant's hand movements during the perturbation phase reflected compensation for the applied perturbation, with hand paths straightening over the rotation block, and also reflected after-effects, with deviations from baseline reaches at the end of a washout period. Faint lines show individual participants’ average hand path during the period, and thick lines show the group average over participants. ***c***, Time course of the mean hand angle averaged over cycles (4 movements). The line shows the average hand angle from the target direction over participants, with the shaded area showing 95% confidence intervals. The labels show periods of cycles over which measures of learning were operationalized. ***d***, Mean hand angles over periods of the experiment. The period labels are abbreviated from those defined in panel ***e***. ***e***, Within-participant difference between early washout and asymptotic learning. For panels ***f*** and ***g***, points show individual participants, bars show the group mean, and vertical bars show the 95% confidence intervals.

To ensure a common analysis procedure was applied for all participants, movement data was resampled to a consistent 100 Hz using linear interpolation and filtered using a second order, zero-phase, low-pass Butterworth filter with a 15 Hz cutoff, with the start and end of each trial's time series padded to avoid transient effects of the filter affecting the movement data. Raw hand paths were visualized (Fig. 2*b*, rotated to a common target and flipped to show a consistent rotation direction) to ensure the actual movements performed by participants were consistent with canonical observations for these task manipulations. To perform quantitative analysis, key measures were extracted from the time series of cursor movements. The main measure used throughout was hand angle at peak speed. To extract this, the peak radial speed reached during the trial was found and the input position at this time was identified (unaffected by perturbations). Hand angle was then defined as the difference in angle between straight lines connecting the start-point to either the target or input position at peak speed. For participants who experienced a counterclockwise perturbation, the sign of the hand angle was flipped so for both perturbation directions, positive hand angles reflected a movement of the hand in the direction opposite to the perturbation. For Experiment 3, hand angles on Inverted Look trials also had an 180° rotation applied so that they reflected the angle from the ideal aim location to make Look and Inverted Look trials directly comparable. Trials in Experiments 1 and 2 were grouped into cycles of four movements (one to each target), with mean hand angles calculated per cycle, whereas for Experiment 3 hand angles were left per trial.

In Experiment 1 we noticed participants occasionally initiated movements to an uncued target, likely because the time pressure applied induced some guessing. For example, at extremely low reaction times in forced response paradigms, participants guess uniformly which target will be probed (Haith et al., 2016). To alleviate the effect of this on our data, we removed trials where the absolute hand angle at both peak speed and take-off (hand angle at a radius of 0.2 au) were >60° from the ideal angle, to ensure the included movements were not directed to an uncued target, which removed 10.5% of trials for the first experiment (inclusion of these trials did not change any statistical analyses). To counteract this, Experiments 2 and 3 had online detection of “jump starts” to ensure any such trials were repeated. This led to 5.3% of trials being repeated in Experiment 2 and 1.6% of trials in Experiment 3 (note the reduction in repeated trials when the time limit was removed for Experiment 3, with a rate comparable with other online adaptation experiments; Avraham et al., 2021; Tsay et al., 2023a). Despite this, some trials did not pass the filter at peak speed, removing 1.2% of trials in Experiment 2 (including these outliers produced identical analyses besides the transfer analysis for Experiment 2b where, despite similar point estimates, there was no statistical difference in transfer between contexts, nor a reduction in learning for the Point-trained group) and 1.6% of trials in Experiment 3 (inclusion of these trials did not change any statistical analyses).

To perform analyses based on periods of the experiment, average hand angles over prespecified periods were calculated. For Experiment 1, where we wanted to understand how hand angle compared between the contexts across the experiment, the baseline period comprised the last 10 cycles of the baseline block, and early and late rotation and washout were defined as the first and last 10 cycles of the rotation and washout blocks, respectively. We also calculated a measure for the after-effect that accounted for potential differences in asymptotic learning by subtracting the hand angle during late learning from that during early washout. For Experiment 2, where we were primarily concerned with transfer, we calculated early and late rotation as in Experiment 1 and additionally calculated transfer as late learning subtracted from the first 10 cycles of the transfer block. For Experiment 3, early and late learning was assessed during the first and last 10 trials during the rotation block, respectively. Analysis of probe trials used baseline-corrected values, where the average hand angle per target in the baseline generalization block was subtracted from average hand angle per target in the generalization block for each participant, to correct for any intrinsic biases (analysis was identical with or without baseline correction).

### Statistical analysis

All statistical analyses were performed in R (version 4.2.2). For all ANOVAs assessing the progression of learning in Experiments 1–3 (performed using the *afex*, *BayesFactor*, and *bayestestR* packages), in addition to reporting the *F* statistic and *p* values, we included Bayes factors (BF_10_) as a measure of the evidence for the alternative hypothesis (H_1_) over the null hypothesis (H_0_) to supplement the *p* values and partial eta squared (*η_p_*^2^) as a measure of effect size. For all follow-up *t* tests using the estimated marginal means (performed using the *emmeans* package) and all independent *t* tests, we use two-tailed tests and report the mean and 95% confidence interval of the differences as well as *p* value and Bayes factor. Finally, for all mixed-effect linear regressions (featuring a random intercept for participant, performed using the *lme4* package with *p* values from the *lmerTest* package calculated according to the Satterthwaite approximation), we report the mean and 95% confidence interval of the regression coefficient. All statistical tests were assessed against a significance threshold of *p* < 0.05.

## Results

To compare participants’ adaptation to perturbations in an FPS-style experiment, we utilized a framework allowing the style of visual feedback to be compared between and within participants (Warburton et al., 2023). Using the Unity game engine, a viewport camera provided a display of a virtual environment, within which task-relevant objects like a start-point and target were located (Fig. 1*a*). A cursor was rendered on top of this display, appearing like an FPS-style aiming reticle, which participants could control using their computer mouse or trackpad (learning did not significantly differ between input devices across all experiments, so is not considered hereafter). Upon left-clicking the start-point, a target immediately appeared, and participants attempted to move to and click on the target.

Input movements could control the cursor in one of two ways (Fig. 1*b*). In the Point context, the in-game camera remained stationary throughout. The start-point and target, therefore, remain stationary on the display throughout a trial, and input movements translated the cursor on top of this static display. This is consistent with how people normally interact with their computer's desktop environment and standard practice for the study of visuomotor adaptation (Cunningham, 1989; Krakauer, 2009; Krakauer et al., 2019; Morehead and de Xivry, 2021). In the Look context, the cursor is always fixed to the center of the camera's display, while input movements are yoked to the in-game camera, panning and tilting its view of the virtual environment. This is consistent with how FPS games are designed, where mouse movements control a character's view of the environment. For example, moving the mouse forward would cause the in-game camera's view to pan up (Movies 1, 2). Given that the participant's display always shows the camera's view, this has the effect of the environment moving down on the display, as if the target is pulled down toward the centrally located cursor. Critically, the two contexts were equated such that the same input movement in either context would reach a given target, allowing unbiased comparison of behavior between the contexts [see Warburton et al. (2023) for details].

### Participants adapt similarly in the point and look contexts

In Experiment 1, we began by assessing whether there were differences in how participants adapted to a perturbation in either context. The visual presentation of the perturbation differed between the contexts, which the participant must use for both online feedback corrections and trial-to-trial adaptation. In the Point context, the cursor moved 30° from the intended direction, whereas in the Look context, the perturbation was applied to the camera's rotation which caused the virtual environment to move 30° from the intended direction across the screen (Fig. 2*a*). During a trial, upon clicking the start-point, a target immediately appeared in one of four locations, located on an imaginary circle in 90° increments. Participants had to move to and click on the target within a time limit to “shoot” it; otherwise, the target would disappear. The time limit was continually staircased throughout the experiment to maintain a ∼50% success rate, inducing the type of time pressure players of FPS games might experience.

Participants first completed a baseline block with veridical movements, showing average movements that were straight to the target (Fig. 2*b*). A visuomotor perturbation was then introduced, where the visual feedback of a movement was rotated around the start position by 30° from the input movement, requiring participants to compensate for this perturbation to be successful. Early movements (first 10 cycles) during this phase showed a large corrective movement near to the target but had straightened by the end of the block (last 10 cycles). Once the perturbation ceased, both groups showed similar “hooked” movements in the opposite direction to overcome after-effects, which were still present by the end of the washout period.

This learning is quantified in the average hand angles (measured at peak speed) per group over the time course of the experiment (Fig. 2*c*). During the late rotation period, where learning appeared to have become asymptotic, participants in each group compensated by 21.3° (Point: 95% CI = 19.4°–23.2°, Look: 19.9°–22.8°). To assess differences over the learning block, hand angles over predefined periods were assessed using a 2 (context, Point vs Look) × 2 (period, early vs late rotation) mixed ANOVA (Fig. 2*d*). The ANOVA showed a main effect of period (*F*_(1, 66)_ = 560.36, *p* < 0.001, BF_10_ > 100, *η_p_*^2^ = 0.90), indicating participants had greater hand angles during late rotation, but no main effect of context (*F*_(1,66)_ = 1.47, *p* = 0.230, BF_10_ = 0.51, *η_p_*^2^ = 0.02). There was, however, a significant interaction between context and period (*F*_(1,66)_ = 4.77, *p* = 0.032, BF_10_ = 1.77, *η_p_*^2^ = 0.07). Participants in the Point group reached a higher compensatory hand angle than those in the Look group during early learning (estimated marginal mean difference [95% confidence interval] = 2.30° [0.46°, 4.15°], *t*_(66)_ = 2.49, *p* = 0.015, BF_10_ = 3.33), but were not significantly different during late learning (−0.03° [−2.46°, 2.40°], *t*_(66)_ = −0.03, *p* = 0.980 BF_10_ = 0.25). We also characterized the after-effect as the difference between early washout and late learning, to account for any potential differences in asymptotic learning that may have arisen (Fig. 2*e*). The decrease in hand angle from late learning to early washout was not significantly different between the Point and Look groups (−0.74° [−3.29°, 1.81°], *t*_(66)_ = 0.58, *p* = 0.565, BF_10_ = 0.29).

### Learning transfers from a trained context to the other untrained context

Overall, behavior in Experiment 1 was strikingly similar between contexts, reaching similar asymptotic levels and decaying in a similar fashion upon removal of the perturbation. The similarity of learning, as well as the similarity across multiple movement metrics in a previous study (Warburton et al., 2023), is consistent with (but insufficient to prove) the use common internal models between contexts. To test this more directly, we trained participants to counteract a perturbation in one context, a process thought to be driven by updates to a forward model and/or controller, before switching to the other, untrained, context. If these contexts do not engage the same adapted internal model, we would expect poor transfer of learning to the untrained context.

In Experiment 2a, participants were assigned a (trained) context to learn to counteract the perturbation in. An experimental trial mimicked Experiment 1 but followed a different block schedule. Participants performed a pair of unperturbed baseline blocks in both the untrained and trained context before learning to counteract a 30° visuomotor rotation in the trained context. To observe the expression of after-effects following a context switch, participants then performed an unperturbed block in the untrained context, before finally performing an unperturbed block in the trained context.

**Figure 3.**
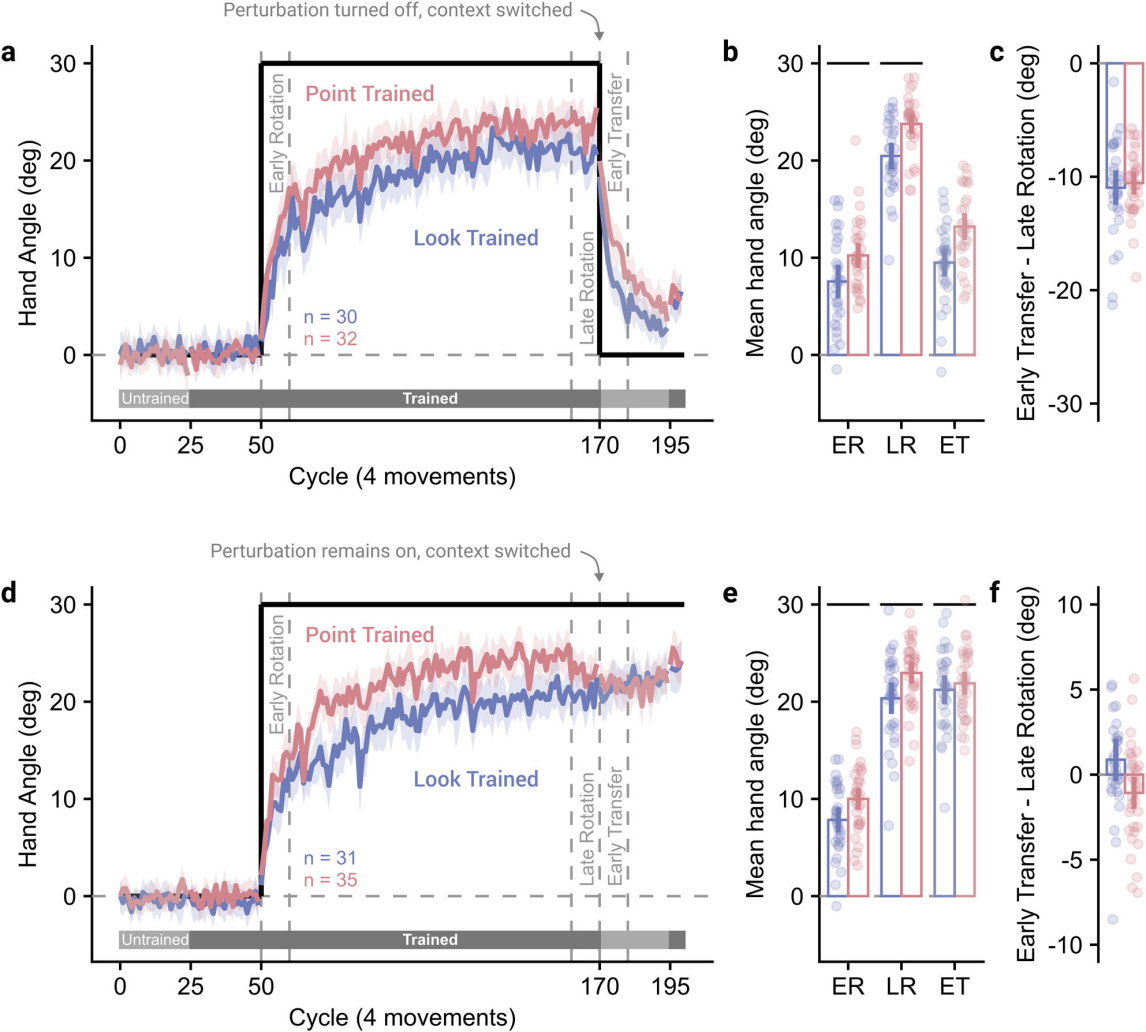
Learning transfers between context. ***a***, Time course of the mean hand angle averaged over cycles (4 movements). The line shows the average hand angle from the target direction over participants, with the shaded area showing 95% confidence intervals. The labels show periods of cycles over which measures of learning were operationalized. Shaded horizontal bars underneath show whether participants were currently completing trials in the trained or untrained context. ***b***, Mean hand angles over periods of the experiment. The period labels are abbreviated from those defined in panel ***a***. ***c***, Within-participant difference between early transfer and late learning. For panels ***b*** and ***c***, points show individual participants, bars show the group mean, and vertical bars show the 95% confidence intervals. ***d-f***, As ***a–c*** with the perturbation left on when switched to the untrained context.

There appeared to be a greater difference between contexts than in Experiment 1, with greater hand angles for the group training in the Point context (Fig. 3*a*). Hand angles during the learning block (Fig. 3*b*) significantly increased between early and late learning (*F*_(1,60)_ = 604.56, *p* < 0.001, BF_10_ > 100, *η_p_*^2^ = 0.91) and were greater for participants trained in the Point context (*F*_(1,60)_ = 13.35, *p* < 0.001, BF_10_ = 51.24, *η_p_*^2^ = 0.18), with no significant interaction (*F*_(1,60)_ = 0.32, *p* = 0.575, BF_10_ = 0.29, *η_p_*^2^ = 0.01).

We next checked for differences in the expression of after-effects. If the learning was bound specifically to the trained context, we would expect a sharp drop in hand angle upon switching to the untrained context. The reduction in hand angle from late learning to early transfer (Fig. 3*c*) was not significantly different between trained contexts (0.41° [−1.45°, 2.27°], *t*_(60)_ = 0.44, *p* = 0.660, BF_10_ = 0.28). Critically, there was no significant difference in the reduction of hand angle when participants switched context, compared with when participants did not switch context in Experiment 1, for either the group trained in the Point context (−1.02° [−3.31°, 1.28°], *t*_(62)_ = 0.88, *p* = 0.380, BF_10_ = 0.36) or Look context (−2.16° [−4.40°, 0.07°], *t*_(64)_ = 1.93, *p* = 0.058, BF_10_ = 1.21). This indicates there was no evidence for a reduction in after-effects when participants switched to an untrained context.

Similar analyses were performed for Experiment 2b, where the perturbation was left on as participants were switched to the untrained context. Hand angles again appeared to be greater for those trained in the Point context (Fig. 3*d*). Hand angles during the learning block (Fig. 3*e*) were again significantly greater during late learning (*F*_(1,64)_ = 557.21, *p* < 0.001, BF_10_ > 100, *η_p_*^2^ = 0.90), and for the participants trained in the Point context (*F*_(1,64)_ = 10.02, *p* = 0.002, BF_10_ = 14.01, *η_p_*^2^ = 0.14), with no significant interaction (*F*_(1,64)_ = 0.18, *p* = 0.674, BF_10_ = 0.27, *η_p_*^2^ < 0.01).

Despite the perturbation being left on in this task, we would still expect a substantial drop in hand angle immediately after transfer if the contexts did not share an internal model. The change in hand angle from late learning to early transfer (Fig. 3*f*) was significantly lower for participants who trained in the Point context compared with the Look context (−1.95° [−3.53°, −0.37°], *t*_(64)_ = −2.47, *p* = 0.016, BF_10_ = 3.18). Participants who trained in the Point context showed a small but significant reduction in hand angle from late learning to early transfer (−1.07° [−2.05°, −0.09°], *t*_(34)_ = −2.23, *p* = 0.032, BF_10_ = 1.56), whereas participants who trained in the Look context showed no significant difference in hand angle between late learning and early transfer (0.88° [−0.43°, 2.19°], *t*_(30)_ = 1.37, *p* = 0.180, BF_10_ = 0.45).

Mixed-effect linear regressions across the untrained transfer block (cycles 170–195) showed that participants trained in the Point context had an intercept significantly lower than asymptotic learning upon switching to the Look context (*β* = −1.28 [−2.33°, −0.24°], *t* = −2.41, *p* = 0.019), with no significant change over cycles (*β* = 0.01 [−0.03°, 0.05°], *t* = 0.53, *p* = 0.599), whereas the intercept did not differ for participants trained in the Look context (*β* = 0.11 [−0.82°, 1.05°], *t* = 0.23, *p* = 0.816) but hand angle did significantly increase over cycles (*β* = 0.07 [0.03°, 0.12°], *t* = 3.15, *p* = 0.002). Both regressions suggest asymptotic learning is slightly lower in the Look context—with those transferred to it experiencing a rapid decrease in hand angle and those transferred from it able to increase their compensatory angle slightly. Nevertheless, both experiments clearly show that the bulk of learning transfers to the untrained context.

### Learning generalizes around the planned movement of the hand

**Figure 4.**
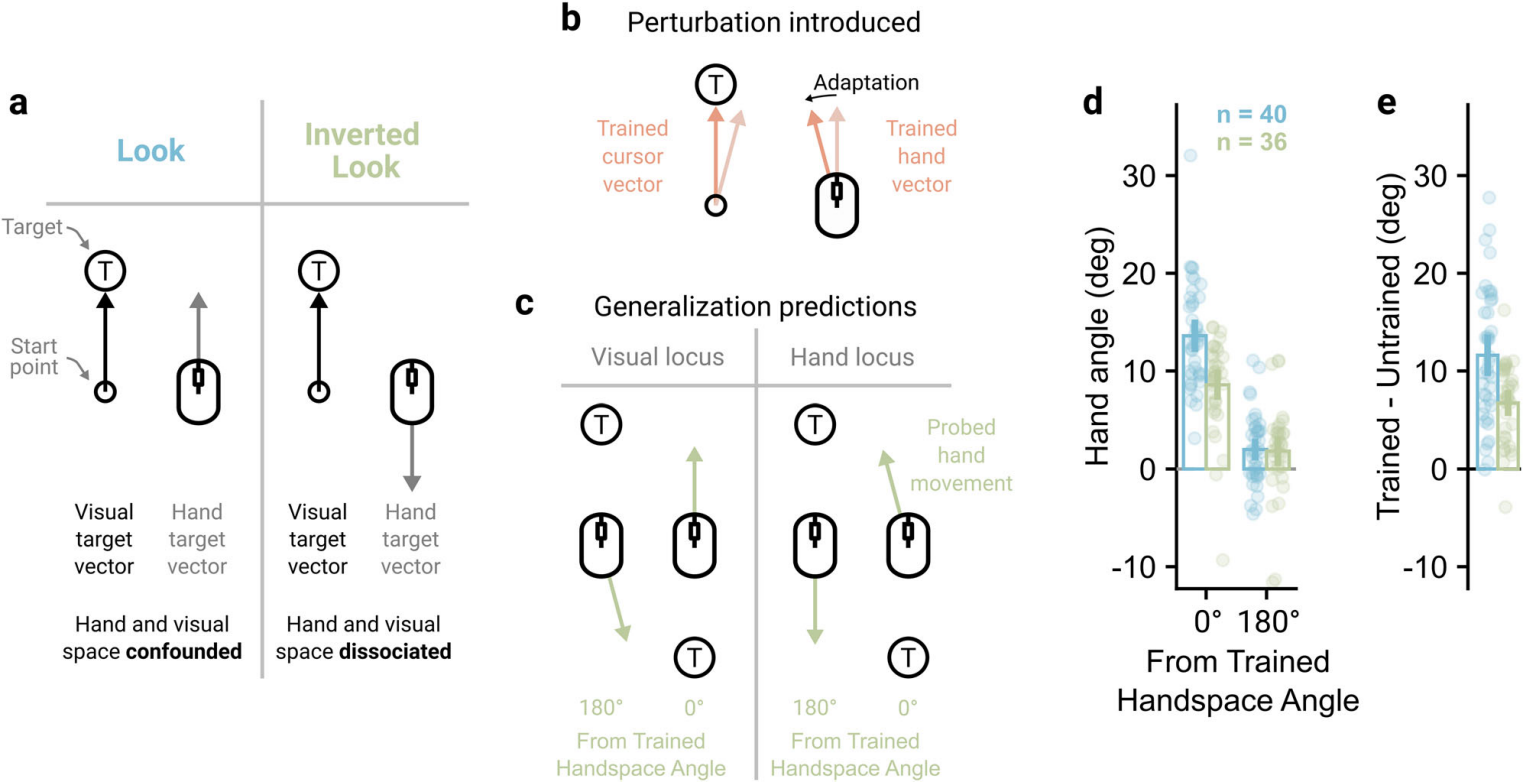
Learning generalizes around the planned hand vector. ***a***, In the Look context, the visual and hand-space vectors between the start-point and target are confounded. Experiment 3 included an Inverted Look context to dissociate them. When participants move their mouse forward, their in-game view pans down. ***b***, Participants learned to counteract a visuomotor rotation at a single target in the Point context, where the planned cursor and hand vectors were common. ***c***, The inverted look allows the planned cursor and hand vectors to be dissociated. A visual locus of learning should show adaptation when the planned cursor vector is congruent with that trained, whereas a hand-based locus should show adaptation when the planned hand vector is congruent with that trained. ***d***, Participants in both groups showed larger amounts of generalization to the trained movement of the hand. ***e***, Within-participant comparisons between trained and untrained movement direction showed larger hand angles for the trained movement. For panels ***d*** and ***e***, bars show mean hand angles and vertical lines show 95% confidence intervals.

The previous experiment found that the bulk of learning transfers between the visual contexts. We suggest this shows the contexts do engage a common adapted internal model and that learning generalizes around a feature common to both tasks. Visuomotor rotations induce learning that generalizes locally around an aiming vector (Day et al., 2016; McDougle et al., 2017). Whether learning is bound to an aim location in visual or motor space is unclear—the two are typically confounded. To investigate this, we used a manipulation taken from the FPS gaming domain, where a subset of games and gamers “invert” their mapping, such that they move their mouse or joystick forward to look down in the game (Fig. 4*a*). The Inverted Look mapping should therefore allow us to dissociate whether learning at a single target (Fig. 4*b*) generalizes locally around the planned vector of the hand, where it would generalize roughly around the trained handspace direction, or the planned vector of the cursor, where it would generalize roughly around the trained visual target (Fig. 4*c*).

**movie 1.** Video demonstrating experimental trials in Experiment 1 during the Baseline and Rotation phases with Look style visual feedback. Participants attempted to move to and click on a target before a time limit to successfully shoot it, otherwise it disappeared. [View online]

**movie 2.** Video demonstrating experimental trials in Experiment 1 during the Baseline and Rotation phases with Point style visual feedback. Participants attempted to move to and click on a target before a time limit to successfully shoot it, otherwise it disappeared. [View online]

In Experiment 3, participants were split into two groups where the spatial generalization of learning was assessed with either Look or Inverted Look probes. Experimental trials using the probed context proceeded as in the previous experiments with two exceptions—there was no time limit, and the upcoming context was signaled by a color cue as participants returned to the start-point. Experimental trials using the Point context were similar except no feedback was provided as participants moved their hand back to the start-point (see Materials and Methods). Following baseline trials to introduce this change in trial feedback, participants completed a baseline generalization block, where 15 generalization cycles were performed. Each cycle had three trials in the probed context, first to either the left or right target and then to both the top and bottom targets, followed by 15 Point trials to the top target. No perturbation was applied during this block, and it allowed the later generalization block to be baseline corrected, removing systematic biases in reaches (Ghilardi et al., 1995).

Participants next experienced 120 Point trials to the top target, where they learned to counteract a 30° visuomotor rotation. Critically, because participants did not have return feedback during these trials, learning should only generalize locally around the aiming vector. Both groups learned to counteract the perturbation similarly, with hand angles increasing between early (10.2°) and late learning (24.2°; *F*_(1,74)_ = 251.69, *p* < 0.001, BF_10_ > 100, *η_p_*^2^ = 0.77), and no significant effect of probed context (*F*_(1,74)_ = 0.96, *p* = 0.331, BF_10_ = 0.35, *η_p_*^2^ = 0.01) or interaction (*F*_(1,74)_ = 0.04, *p* = 0.849, BF_10_ = 0.24, *η_p_*^2^ < 0.01).

Participants then completed the generalization block, which was identical to the baseline generalization block except that the 15 Point trials per cycle had the 30° visuomotor rotation applied. Because unlearning will occur during the unperturbed probe trials, enough relearning trials (established in a pilot study) were allowed to “top up” learning for the top target (average hand angle over last 5 top up trials = 26.2°). Taking just the probe trials in the same and opposite direction to the trained handspace direction (Fig. 4*d*), a significant main effect of angle from the trained handspace direction indicated learning generalized around the planned hand vector (*F*_(1,74)_ = 197.04, *p* < 0.001, BF_10_ > 100, *η_p_*^2^ = 0.73). Further, there was a main effect of probed context (*F*_(1,74)_ = 9.64, *p* = 0.003, BF_10_ = 9.66, *η_p_*^2^ = 0.12), indicating hand angle was greater for the Look probes, and a significant interaction (*F*_(1,74)_ = 13.88, *p* < 0.001, BF_10_= 95.26, *η_p_*^2^ = 0.16). This interaction was followed up by assessing the differences in hand angle between trained and untrained movement directions. Hand angle was greater for the trained handspace direction compared with the untrained handspace direction in both Look (11.61° [9.81°, 13.40°], *t*_(74)_ = 12.90, *p* < 0.001, BF_10_ > 100) and Inverted Look groups (6.74° [4.85°, 8.63°], *t*_(74)_ = 7.11, *p* < 0.001, BF_10_ > 100; Fig. 4*e*). Therefore, both groups show a robust transfer of learning from the trained Point context to the probed Look or Inverted Look context, supporting the previous experiment's results, and the results are consistent in that learning generalizes around planned movement of the hand.

This difference was, however, significantly greater for the Look group compared with the Inverted Look group (4.87° [2.26°, 7.47°], *t*_(74)_ = 3.73, *p* < 0.001, BF_10_ = 69.94). A mixed-effect linear regression, assessing how hand angle for the trained input direction changed over generalization cycle, showed there was no significant difference between probed context on the first generalization cycle (*β* = −2.17 [−5.02°, 0.69°], *t* = −1.48, *p* = 0.140). The Look group showed no significant change in hand angle over cycles (*β* = 0.04 [−0.12°, 0.20°], *t* = 0.45, *p* = 0.651), but the Inverted Look had a significant decrease in hand angle over cycles (*β* = −0.29 [−0.53°, −0.05°], *t* = −2.40, *p* = 0.017). Therefore, while hand angle is not significantly lower for the Inverted Look group at the start of the generalization block, the groups diverged over the cycles. This could indicate that, for the Inverted Look group, transferred learning itself reduced over cycles, which could happen if the number of top-up trials was not enough for the group to re-establish a consistent amount of implicit learning or that participants formed an explicit strategy to overcome the implicit learning shown in the trained input direction, which was possible given participants had full vision during the probe trials.

## Discussion

Here we examined transfer of visuomotor adaptation across two task contexts where a common input movement drove categorically distinct visual feedback. We found that adaptation occurred similarly in both contexts and transferred readily between them. Additionally, we showed that learning generalizes around the planned movement of the hand, rather than specific visual features of the task. These results demonstrate that visuomotor adaptation modifies an element of the visuomotor control circuit that is downstream of the specific features of the visual feedback. We believe these results have meaningful implications for the theory of internal models, particularly regarding the role of sensory feedback in motor control.

### Visual contingencies

Following our previous work, which demonstrated similar goal-directed movements were elicited in response to two styles of visual feedback delivering motion energy in opposite directions (Warburton et al., 2023), we found that visuomotor adaptation is not critically tied to such categorical differences in movement-related visual motion. Learning progressed similarly between visual contexts in the first experiment, with both groups showing a comparable reduction in hand angle once the visuomotor rotation ceased. In Experiment 2, we showed that learning in one visual context transfers almost entirely to the other, untrained, visual context. The third experiment further demonstrated transfer of learning from the trained Point context to untrained Look and Inverted Look contexts. While there is debate around the error signal important for visuomotor adaptation (Tseng et al., 2007; Kim et al., 2019; Tsay et al., 2022), and what changes this error drives (Shadmehr et al., 2010; Hadjiosif et al., 2021), we find that the same motor memories are engaged and altered by these different forms of visual feedback.

### Internal models

Internal models are ubiquitous in modern theories of motor control and learning (Shadmehr and Krakauer, 2008; Krakauer et al., 2019; McNamee and Wolpert, 2019). Both controllers (inverse models) and forward models require continuous calibration to maintain skilled moment, but there is ongoing debate as to which primarily drives adaptation. Where jointly assessed, learning in a forward model appears to be relatively more important (Bhushan and Shadmehr, 1999). Indeed, updates to a forward model driving adaptation has emerged as a popular hypothesis (Shadmehr et al., 2010; Krakauer and Mazzoni, 2011; Haith and Krakauer, 2013), with sensory prediction errors thought to be the key error signal (Mazzoni, 2006; Tseng et al., 2007). If updates to a forward model drive adaptation, our current results would suggest that sensory predictions must be made at a sufficiently abstract level for either visual context to engage the same adapted forward model. Notably the relative motion between cursor and target is the same at any given point in time between the two visual contexts. This means that displacement vectors between cursor and target in visuospatial coordinates would constitute a sufficiently abstracted level of visual representation for sensory predictions.

Alternatively, adaptation may be driven by updates to the controller (or inverse model). Some putative neural architectures suggest the involvement of forward models (e.g., a forward model may be used to convert performance errors into motor errors to train an inverse model; Jordan and Rumelhart, 1992; Miall and Wolpert, 1996; Flanagan et al., 2003). In contrast, performance errors in direct policy learning (Hadjiosif et al., 2021) or corrective movements in feedback-error learning (Kawato and Gomi, 1992; Albert and Shadmehr, 2016) could be used to train the controller without the requirement of a forward model. Under this scenario, our results would suggest that the input to the inverse model must cover both visual scenarios. In some formulations this input is a desired sensory state (Jordan and Rumelhart, 1992), which could again be cursor–target displacement vectors in visuospatial coordinates. Other formulations have a desired trajectory or “motor plan” as the input to the inverse model (Kawato and Gomi, 1992), in which case the translation of a desired sensory change to an abstract motor plan must occur upstream. In either case, our results would suggest that more direct representations of sensory outcomes are not relevant to the motor system.

The outlined forward and inverse model adaptation scenarios are consistent with research demonstrating a critical role for movement vectors in motor control and learning. The effector-centered direction and extent of a movement appear to be key parameters, specified separately during planning (Rosenbaum, 1980; Favilla et al., 1989; Gordon et al., 1994; Vindras and Viviani, 1998). Further, the movement vector appears to be the predominant feature remapped during adaptation (Krakauer et al., 2000; Wang and Sainburg, 2005; Wu and Smith, 2013), with spatial generalization greatest in the direction participants aimed toward (Day et al., 2016; McDougle et al., 2017; Kim et al., 2022). By dissociating hand and cursor movements in Experiment 3, we showed that the locus of learning is specifically the planned movement vector of the hand, rather than a visually referenced aim location. This places the hand space vector between effector and goal as a common currency for the motor system. This is consistent with the hypothesized role of the posterior parietal cortex as a sensorimotor interface which encodes effector-target vectors in multiple coordinate frames (Buneo and Andersen, 2006) and work suggesting the connection between the posterior parietal cortex and premotor areas as an adaptation locus (Tanaka et al., 2009).

In principle the two visual contexts could engage distinct internal models and distribute learning to each other through a mixture-of-experts approach (Wolpert and Kawato, 1998; Haruno et al., 2001). However, this would require that participants already had the requisite internal models for the Look context. Only 19 of 68 participants in Experiment 1 reported playing FPS games, with 14 reporting playing no games at all, and tasks thought to elicit de novo acquisition of a new internal model show extended periods of poor performance (Haith et al., 2022). It therefore seems unlikely that participants with little to no experience with FPS games could acquire such internal models and switch between them so rapidly.

We expect that the learning observed reflects both implicit and explicit processes (Taylor et al., 2014; McDougle et al., 2016). Our conclusions about internal models are focused on the implicit component. In cases where learning is explicit and cued by context, strategies can be rapidly disengaged (Morehead et al., 2015, 2017; Tsay et al., 2023b; Chen et al., 2024), whereas in Experiment 2a we found similar aftereffect profiles whether participants did or did not switch context. Further, in Experiment 3 we observed generalization of learning from a trained Point context to untrained Look and Inverted Look contexts. The reduction in hand angle from top-up training trials to probe trials would suggest the modulation of an explicit strategy. However, the fully cued nature of the probe trials, along with the lack of substantial generalization to untrained directions, is consistent with the probe trials sampling internal model adaptation. As such, we believe our results can only be explained by a shared adapted internal model.

### Contextual cues

Recent work has found contextual cues that allow multiple visuomotor maps to be learned concurrently. Primarily these are cues relevant to the movement itself, for example, the physical workspace location (Hwang et al., 2006; Howard et al., 2013), planned movement (Hirashima and Nozaki, 2012), or (planned) lead-in or follow-through movement (Howard et al., 2012, 2015; Sheahan et al., 2016; Dawidowicz et al., 2022). However, some visual cues, like the visual workspace location (Howard et al., 2013) or participant gaze location (Abekawa et al., 2022), also appear effective. Thus, while we found that learning transferred between visual contexts by default, these visual contexts may serve as effective contextual cues. Indeed, many gamers express an Inverted Look mapping in an FPS game, moving their mouse forward to reach a target directly down, yet switch to typical Point movements when moving their cursor in other non-FPS games. Future research could, therefore, look at the time scale and cues necessary to allow opposing mappings to be retained long term and contextually switched in this manner.

### Studying learning in FPS games

Video games offer an exciting opportunity to study situated skilled behavior. For example, data collected from a commercial FPS aim-trainer game have been used to investigate long-term skill acquisition (Listman et al., 2021) and the kinematic correlates of skill (Donovan et al., 2022). Such approaches enable a player's game skill to be tracked (Stafford and Dewar, 2014; Stafford and Vaci, 2022), offering insights about long-term motor learning that are difficult to make with restricted laboratory studies. Movement kinematics have also been used to study the dynamics of perceptual and cognitive processes (Spivey and Dale, 2006; Song and Nakayama, 2009; Freeman et al., 2011; Dotan et al., 2019). Given that FPS games typically engage a range of perceptual, cognitive, and motor abilities (Green and Bavelier, 2012), the kinematic data recorded in gameplay may provide unprecedented insights into situated skill. Our current and previous findings (Warburton et al., 2023) suggest that movement and learning properties are largely invariant to the differences in visual feedback between FPS games and traditional laboratory setups, which should give researchers the confidence to utilize this exciting medium to study skilled behavior.

## Data Availability

All data and scripts to analyze the data are available at https://osf.io/8s9mk/.
